# Experiences of Women With Medical Abortion Care Reflected in Social Media (VEILLE Study): Noninterventional Retrospective Exploratory Infodemiology Study

**DOI:** 10.2196/49335

**Published:** 2024-05-02

**Authors:** Giulia Gouy, Luisa Attali, Paméla Voillot, Patrick Fournet, Aubert Agostini

**Affiliations:** 1 Service de Gynécologie-Obstétrique Hôpital de la Croix-Rousse Lyon France; 2 Pôle de Gynécologie-Obstétrique et Fertilité Hôpital de Hautepierre Centre Hospitalier Universitaire de Strasbourg Strasbourg France; 3 Kap Code Paris France; 4 Centre de Santé SoMeD Rouen France; 5 Service de Gynécologie et d’Obstétrique Hôpital de la Conception Assistance Publique-Hôpitaux de Marseille Marseille France

**Keywords:** infodemiology, medical abortion, patient experience, real-world evidence, social media, abortion, women's health, reproduction, reproductive, obstetric, obstetrics, gynecology, gynecological, text mining, topic model, topic modeling, natural language processing, NLP

## Abstract

**Background:**

Abortion (also known as termination of pregnancy) is an essential element of women’s reproductive health care. Feedback from women who underwent medical termination of pregnancy about their experience is crucial to help practitioners identify women’s needs and develop necessary tools to improve the abortion care process. However, the collection of this feedback is quite challenging. Social media offer anonymity for women who share their abortion experience.

**Objective:**

This exploratory infodemiology study aimed to analyze, through French social media posts, personal medical symptoms and the different experiences and information dynamics associated with the medical abortion process.

**Methods:**

A retrospective study was performed by analyzing posts geolocated in France and published from January 1, 2017, to November 30, 2021. Posts were extracted from all French-language general and specialized publicly available web forums using specific keywords. Extracted messages were cleaned and pseudonymized. Automatic natural language processing methods were used to identify posts from women having experienced medical abortion. Biterm topic modeling was used to identify the main discussion themes and the Medical Dictionary for Regulatory Activities was used to identify medical terms. Encountered difficulties were explored using qualitative research methods until the saturation of concepts was reached.

**Results:**

Analysis of 5398 identified posts (3409 users) led to the identification of 9 major topics: personal experience (n=2413 posts, 44.7%), community support (n=1058, 19.6%), pain and bleeding (n=797, 14.8%), psychological experience (n=760, 14.1%), questioned efficacy (n=410, 7.6%), social pressure (n=373, 6.9%), positive experiences (n=257, 4.8%), menstrual cycle disorders (n=107, 2%), and reported inefficacy (n=104, 1.9%). Pain, which was mentioned in 1627 (30.1%) of the 5398 posts by 1024 (30.0%) of the 3409 users, was the most frequently reported medical term. Pain was considered severe to unbearable in 24.5% of the cases (399 of the 1627 posts). Lack of information was the most frequently reported difficulty during and after the process.

**Conclusions:**

Our findings suggest that French women used social media to share their experiences, offer and find support, and provide and receive information regarding medical abortion. Infodemiology appears to be a useful tool to obtain women’s feedback, therefore offering the opportunity to enhance care in women undergoing medical abortion.

## Introduction

### Background

Abortion is a common procedure. Worldwide from 2015 to 2019, there were 121.0 million unintended pregnancies annually leading to 73.0 million abortions (60%) [1]. In France, in 2022, there were 234,300 abortions, 78% of which were medical termination of pregnancy (MToP) that is usually performed early (<8 weeks of amenorrhea) [2].

Early MToP with an antiprogestin (ie, mifepristone) followed by a prostaglandin analog has revolutionized abortion. Since mifepristone was first approved in 1988, MToP has been authorized in numerous countries worldwide [3], and its practice has followed changes in health requirements and local regulations. For instance, the COVID-19 pandemic and associated mandatory lockdowns increased the overall rate of MToPs and the rate of MToPs performed at home [4]. Abortion was legalized in France on January 17, 1975 [5], and mifepristone was first approved for MToP in 1988. The current dose regimen for mifepristone and a prostaglandin analog was approved in 2007 [6]. The sequence of consultations includes a first visit to inform the woman, a second visit during which she signs a consent form and initiates the procedure, and a follow-up visit 14 to 21 days after mifepristone intake to ensure the success of the procedure [7].

Abortion is not perceived in the same way as other standard medical acts. In France, until 2001, a motivational interview was mandatory during the first visit. This allowed a survey showing that abortion was still considered to be too much of a taboo to be performed. Women indicated having elaborate defense strategies to protect themselves against this stigma, including keeping their abortion secret, leading to potential unsafe abortion practices [8]. Currently, women can generally express their abortion experience more freely. In particular, social media provide online anonymity and offer a safe opportunity for women to share and seek information.

With over 2.3 billion active users globally [9], social media have become a new data source for public health, as users can find, exchange, and discuss health information on the platforms. According to Médiamétrie, a company specializing in audience measurement and the study of the use of audiovisual and digital media, more than 85% of the French population are internet users [10]. Moreover, according to a recent report [11], 21.1% of French individuals frequently or very frequently use health-related social media such as Doctissimo, with 14.1% using Facebook and 9.8% using women’s magazines and their associated websites to find health information.

Analyzing online information represents a developing alternative means to understand patients’ health compared with self-administered questionnaires. These patient-generated health data are produced spontaneously (and are thus not limited to medical consultations, for instance), mostly anonymously. Therefore, these data may better correspond to patients’ feelings compared with closed-ended questions. Moreover, text-mining techniques applied to analyze social media data can be used with relative ease [12], providing new opportunities to bridge the gap between qualitative and quantitative data analyses [13]. A new research discipline and methodology has thus emerged. This scientific discipline called infodemiology focuses on health-related content analysis published online [14].

Studies based on analysis of Instagram, Facebook, or Reddit posts have started to be published in peer-reviewed journals. These analyses have helped to characterize patients’ experiences and related perceptions of an illness and its burden in many health fields (eg, in vitro fertilization [15], miscarriage [16], cesarean section [17], and breast cancer [18]), along with documenting the processes involved in abortion method decision-making [19]. However, to the best of our knowledge, few studies using social media data have been published to characterize the experiences and perceptions of women who underwent an abortion, in particular MToP [19]. Furthermore, none of these studies has been carried out in France, although women’s experiences are likely influenced by clinical practices and cultural differences that preclude the generalization of data collected in other countries.

### Objective

We conducted VEILLE, a 4-year retrospective infodemiology study, to analyze reported medical symptoms and the different experiences and information dynamics associated with the MToP process in France.

Abortion care is an essential element of women’s reproductive health care [20]. Women's feedback about their experience is crucial to meeting women’s needs during MToP. Collecting women’s feedback about their MToP experiences and understanding these experiences could provide the necessary information for health care providers to respond to French women’s needs during MToP. 

## Methods

### Study Design

VEILLE is a noninterventional, retrospective study using a text-mining approach to retrieve and analyze medical abortion posts from social media posts.

All messages geolocated in France posted by women who had experienced MToP between January 1, 2017, and November 30, 2021, in French-speaking general and specialized web forums were considered. Only messages from publicly available sources were extracted.

The study name VEILLE is an allusion to the French translation of social media monitoring (*veille*) and a tribute to Simone Veil (same pronunciation, /vɛj/) who legalized abortion in France.

### Data Extraction

Data (verbatim social media posts) were identified and pseudonymized by tokenization before being extracted. Irrelevant material was eliminated.

All public posts available on the web containing at least one of the relevant keywords related to MToP were identified using the Brandwatch social media data extractor [21]. This tool is based on queries that include selected keywords evocative of the subject of interest. Using the query, the Brandwatch extractor searches through available public data sources and identifies keywords within posts matching those in the query.

Posts were downloaded along with their associated metadata: URL/domain, publication date, forum, language used, hashtags, authors, and engagement type such as retweet. Posts and associated metadata constituted the corpus.

Keywords in French (eg, IVG for *interruption volontaire de grossesse*, voluntary pregnancy termination) and their synonyms were defined by the authors (see Multimedia Appendix 1).

### Data Preprocessing and Modeling

Extracted posts were cleaned before being stored in the study-specific database. Posts from irrelevant sources such as potential advertising sites or forums related to pets and animals were removed using regular expression rules. Duplicates were managed by merging posts with either the same username on different platforms or the same post with another username. A machine-learning algorithm (extreme gradient boosting classifier [XGBoost]) was used to identify posts reporting personal experiences [22]. These posts constituted the study data set.

The algorithm was implemented based on message-level calculation of the user’s probability of being a woman having experienced MToP according to specific features (lexical fields and regular forms, such as “I have [EXTRACTION TERM]”) and coupled with pronoun variables. Filters (in French) were used to narrow down the search to only MToP experience, excluding surgical abortion (Multimedia Appendix 2).

### Data Analysis

Descriptive analysis was performed for posts (number and source) and social media users (number, age, gender). A social media user’s age was determined through the identification of regular expressions such as “*j’ai 45 ans*” (“I am 45 years old”), “*ayant 45 ans*” (“being 45”) (Regex method) over all posts. Each pseudonym was associated with one gender (man, woman, or unidentified) and one age category (20 years or younger, 21-30 years, 31-40 years, and so on, or unidentified). Gender was confirmed using the Regex method and with the support vector machine algorithm (XGBoost method) through the identification of regular expressions in the content of each post: gendered participles, adjectives, and names (eg, Miss, pregnant) or grammatical features [23].

A *topic model* was applied to identify the topics addressed in the posts constituting the study data set [24]. Topic models consist of text-mining approaches that aim to automatically identify the abstract topics addressed in a collection of documents. Such models are based on the hypothesis that each document corresponds to a distribution of several topics.

A biterm topic model (BTM) was used to identify the topics without prior knowledge. A topic is defined as a subject of discussion, which amounts to tokens that frequently appear together in the posts from the data set. The BTM considers the whole data set as a mixture of topics, where each co-occurring pair in tokens (the biterm) is drawn from a specific topic independently and modeled topics are probability distributions over the biterms [24]. As topics are probability distributions over tokens of the study data set, they can be characterized by the highest per-topic probability tokens. Weighting these probabilities through term-frequency inverse document frequency (TF-IDF) weighting allows topic-specific tokens to be allocated with higher importance. In this case, the per-topic probability of a token is weighted by the inverse of the probabilities of this token in other topics. Therefore, for each topic, tokens were ranked from the highest to the lowest weighted probabilities TF-IDF value in this topic. The first 9 tokens were designated as the set of characteristic tokens and used to name the topic manually.

A specific list of symptoms related to MToP was established based on the Medical Dictionary for Regulatory Activities (version 23) terms [23]. The lexical field was enriched to consider verbal forms found on social networks. A single post could contain several medical terms.

For *difficulties* encountered by social media users, posts were randomly allocated to create a sample representing 30% of the extracted posts. A qualitative manual search was performed on this sample using a generic annotation grid, which helped to categorize each difficulty. A single post could contain several encountered difficulties. Given the diversity of encountered difficulties, data saturation was used to obtain a representative sample of expressed difficulties [25].

Saturation was checked by taking 5% samples of the total number of social media users (N=1964) and analyzing the number of new types of difficulties or unmet needs per 5% sample (n=98). Saturation was considered to be achieved when two consecutive samples no longer yielded more than one newly identified difficulty category. Two additional batches of 5% each were analyzed after saturation was first reached for further validation of the findings [25].

### Ethical Considerations

Data collection and treatment followed the European Union General Data Protection Regulation. The study was conducted within the frame of legitimate interest. The study involved data issued from publicly available sources. Consent was not required as the study involved publicly available posts and as users automatically grant their consent for the reuse of their data when they post on public platforms. Following this and as this falls under the R1121-1 Article of the French Public Health Code [26] (in effect since July 1, 2021), we did not seek ethics board review or approval for this study. Private groups or web pages were excluded from our data extraction process. The results of the study do not contain any identifiable information and are presented taken together. A privacy-by-design approach was adopted as all usernames, web forum names, geographic locations, URLs, or any other sensitive information was substituted by identifiers before being stored in the analysis corpus.

## Results

### Population and Posts

**Figure 1:**
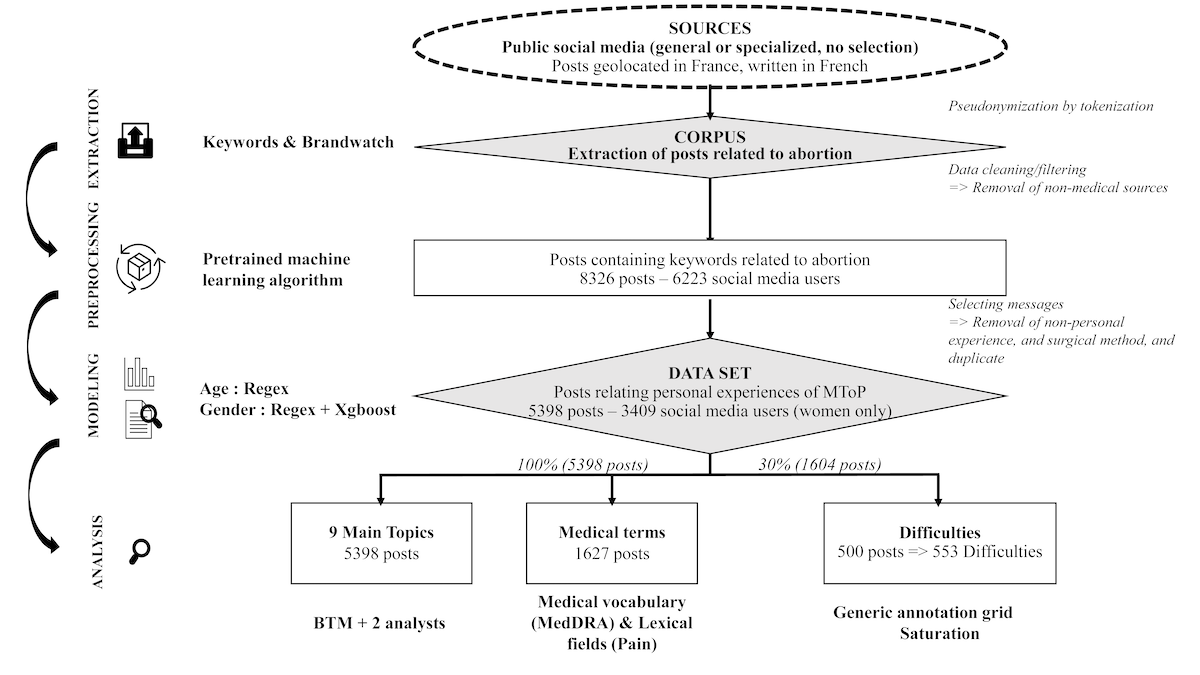
Study framework and flowchart of data extraction and analysis. BTM: biterm topic model; MedDRA; Medical Dictionary for Regulatory Activities; Xgboost: extreme gradient boosting.

After cleaning/filtering of the corpus, 8326 posts published by 6223 users were extracted from social media platforms to be preprocessed/modeled. Therefore, 5398 posts published by 3409 social media users were identified and constituted the study data set (Figure 1). The top 6 keywords are presented in Table 1.

As only posts reported by individuals having experienced MToP could be included in the data set, all posts were deemed to be written by women. Gender was confirmed for 2898 of the 3409 (85%) social media users. Age was found in the posts of 8.1% of the social media users (n=275): 1.5% (n=52) were ≤20 years, 4.2% (n=142) were between 20 and 30 years, 1.5% (n=51) were between 30 and 40 years, and 0.9% (n=30) were >40 years. The median age was 26 years.

The 5398 posts were retrieved from a total of 22 web forums (Table 1); 78% of the posts were issued from two specialized forums (Doctissimo and aufeminin.com) and one general forum (Facebook). Doctissimo, which was the top-ranked source is a French specialized medical site, whereas aufeminin.com, ranking second, is an online women’s magazine. The remaining sources (22%) were specialized (ie, women, patient, or disease-driven) web forums, except for Twitter, Reddit, and YouTube.

**Table 1:** List of forums reporting women’s medical termination of pregnancy experiences and the top 6 extraction keywords.

Feature			Posts (N=5398), n (%)	Social media users (N=3409), n (%)
**Keyword extraction (top 6)**				
	IVG^a^/IVG medicament^b^		3642 (67.5)	N/A^c^
	Avort^d^		761 (14.1)	N/A
	GYMISO		350 (6.5)	N/A
	MIFEGYNE/MIFEGINE		211 (3.9)	N/A
	MISOPROSTOL		188 (3.5)	N/A
	MISOONE		111 (2.1)	N/A
**Forums**				
	**Specialized forums**			
		Doctissimo	2218 (41.1)	1018 (29.9)
		AuFeminin	1001 (18.5)	768 (22.5)
		Journal des femmes	353 (6.5)	241 (7.1)
		Babycenter.fr	336 (6.2)	254 (7.5)
		Journaldesfemmes.com	86 (1.6)	58 (1.7)
		Mamanandco	57 (1.1)	38 (1.1)
		Mademoizelle.com	16 (0.3)	15 (0.4)
		Magicmaman	15 (0.3)	15 (0.4)
		Enceinte.com	14 (0.3)	14 (0.4)
		Psychologies	13 (0.2)	13 (0.4)
		Fiv.fr	10 (0.2)	10 (0.3)
		Beauté test	9 (0.2)	9 (0.26)
		Parents.fr	5 (<0.1)	5 (0.15)
		Thyroide	2 (<0.1)	2 (<0.1)
		Lymphome espoir	2 (<0.1)	2 (<0.1)
		Notrefamille	2 (<0.1)	2 (<0.1)
		Alexia.fr	2 (<0.1)	2 (<0.1)
		Entrepatients.net	1 (<0.1)	1 (<0.1)
	**General forums**			
		Facebook	993 (18.4)	743 (21.8)
		Twitter	174 (3.2)	136 (4.0)
		Reddit	55 (1.0)	34 (1.0)
		YouTube	34 (0.6)	29 (0.9)

^a^IVG: French abbreviation for voluntary termination of pregnancy.

^b^medicament: French word for medicine.

^c^N/A: not applicable.

^d^Avort: first letters of the French word *avortement,* which means abortion.

### Discussion Topics

From the 5398 posts, 9 topics of interest were identified (Table 2). Personal experience and community support were the most prominent topics. The 7 other topics were as follows (in decreasing order): pain and bleeding, psychological experience, questioned efficacy, social pressure, positive experiences, menstrual cycle disorders, and reported inefficacy.

**Table 2:** Topics and topic description ranked by frequency.^a^

Rank	Topic	Posts (N=5398), n (%)	Description
1	Personal experience	2413 (44.7)	Users shared personal experiences. They described what they experienced during their medical abortion, the details of the procedure, and what they felt at that moment.
2	Community support	1058 (19.6)	Looking for community support. Some users looked for experiences shared by other users about the procedure to increase their knowledge and to be prepared for it, as well as to feel reassured.
3	Pain and bleeding	797 (14.8)	Seeking for testimonies about pain and bleeding. Highlighted a lack of information on these drug-related adverse events. Users were concerned about what they were about to experience, and they found nonreassuring testimonies on social media.
4	Psychological experience	760 (14.1)	Users expressed regrets and mental outcomes such as depression and emotional distress with short- and long-term consequences. They also reported that medical abortion was “traumatic” and that if they had known they would have chosen surgical abortion.
5	Questioned efficacy	410 (7.6)	Efficacy was questioned.
6	Social pressure	373 (6.9)	The pressure was from the family and mostly from the partner.
7	Positive experience	257 (4.8)	Shared positive experiences with medical abortion.
8	Menstrual cycle disorders	107 (2.0)	Some users reported menstrual cycle disorders following abortion.
9	Reported inefficacy	104 (1.9)	Some users reported inefficacy of the procedure (medical abortion).

^a^A single post may contain several topics.

### Medical Terms

Pain was the most frequently reported medical term related to difficulties (Table 3). Bleeding was the second most frequent medical term. Pain and bleeding were reported both during and after medical abortion. Other medical terms reported during and after medical abortion were nausea or vomiting (475/5398, 8.8%) and fatigue. Stress and anxiety were directly associated with the medical abortion procedure, including the fear of abortion inefficacy. Emotional distress, echography, and delayed menstruation were reported both before (when pregnancy was confirmed) and after medical abortion. After the procedure, emotional distress was associated with the feeling of regret and grief.

**Figure 2:**
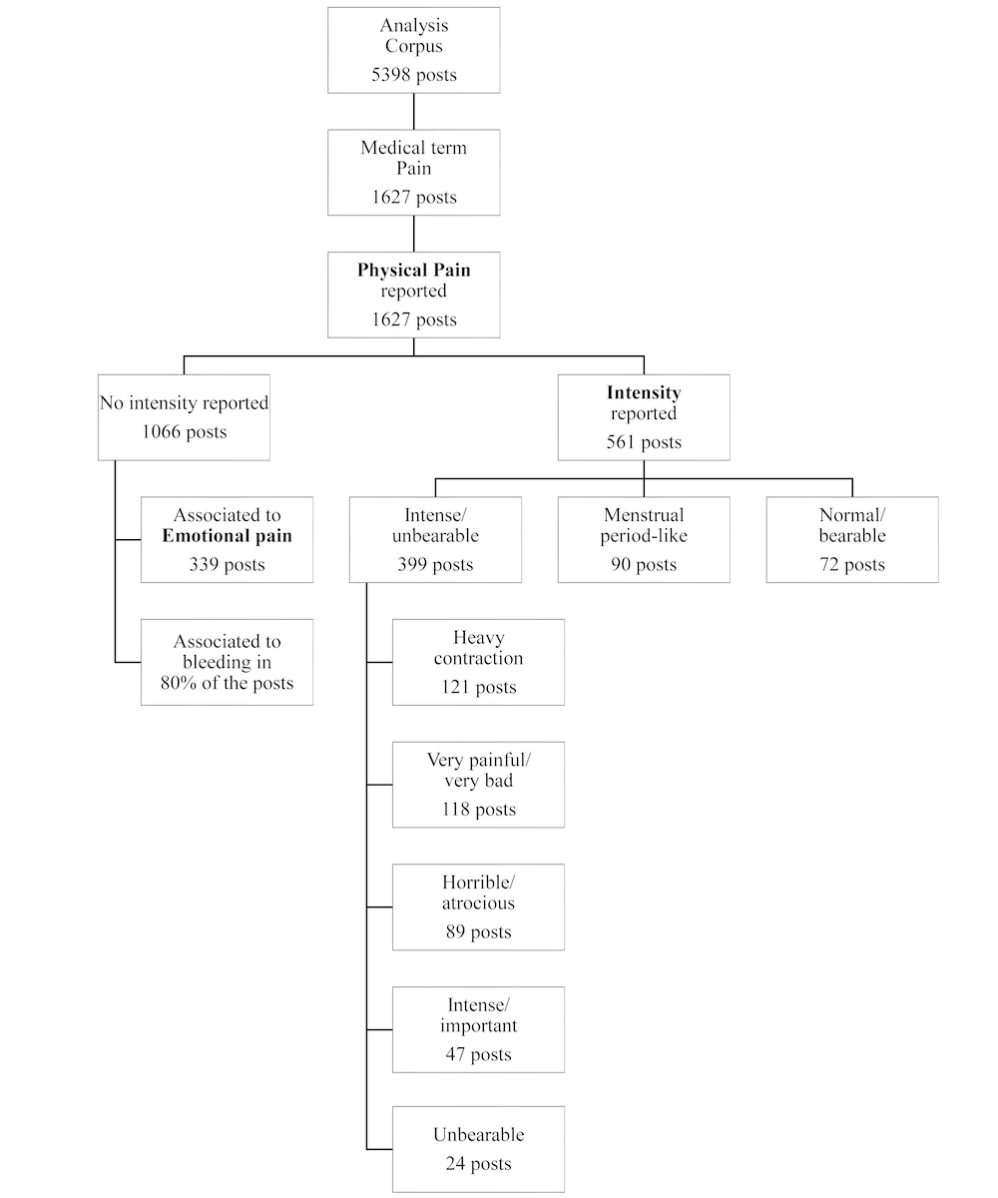
Focus on pain medical terms.

Pain is a multimodal concept with subjectivity, which was reported by 1024 of the 3409 (30.3%) users in 1627 of the 5398 posts (30.1%). Pain usually occurred after the second drug intake (prostaglandin analogs). Using topic modeling, different types of pain were identified, providing details to characterize each type. As a result, two main types of pain were identified: physical and emotional pain. Of the 1627 posts regarding physical pain, the pain intensity was described in 561 posts (34.5%) and was considered severe to unbearable in 399 posts (24.5%) (Figure 2). These rates are not associated with new safety signals in MToP.

**Table 3:** Most frequently reported medical terms related to difficulties after medical termination of pregancy.^a^

Rank	Medical term	Posts mentioning term (N=5398), n (%)
1	Pain	1627 (30.1)
2	Bleeding	1112 (20.6)
3	Emotional distress	997 (18.5)
4	Echography	492 (9.1)
5	Stress/anxiety	460 (8.5)
6	Fatigue	360 (6.7)
7	Nausea	252 (4.7)
8	Vomiting	223 (4.1)
9	Delayed menstruation	134 (2.5)
10	Grief	114 (2.1)

^a^A single post may contain several medical terms.

### Encountered Difficulties

**Figure 3:**
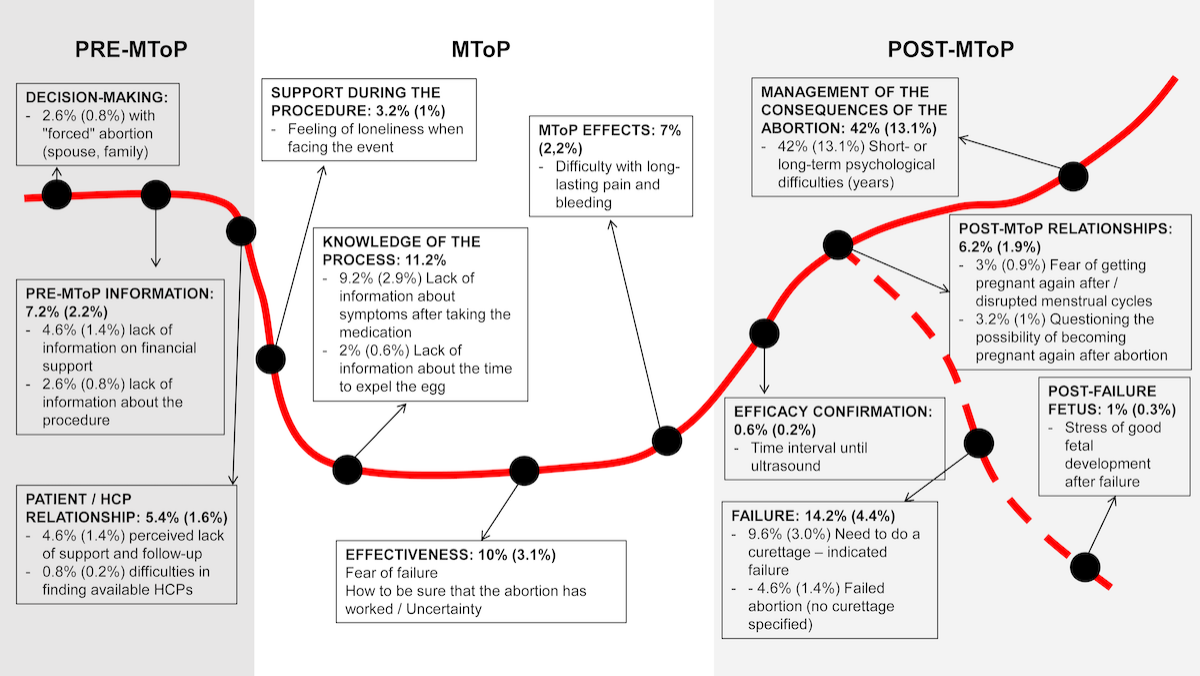
Encountered difficulties. The red line represents the pathway from premedical MToP to post-MToP via MToP. The dashed red line represents the pathway at the end of the procedure in case of failure. Encountered difficulties are summarized in boxes distributed along the whole pathway. HCP: health care professional; MToP: medical termination of pregnancy; x% (y%): number of difficulties among the 553 identified difficulties (number of difficulties among the 1604 posts of the randomized sample).

A total of 553 difficulties were identified from 500 posts derived from a randomized sample containing 1604 posts (30%). Reported difficulties were encountered along the MToP process (Figure 3).

Lack of information was reported within each stage of the MToP process (before, during, and after medical abortion). Before the procedure, users enquired about reimbursement and the MToP process in detail (ie, drug dosage, route of administration). Details about the MToP process were still misunderstood during the process as users were asking questions about the potential side effects, delay of action, and monitoring. After taking the medications, users were concerned about pain and bleeding, especially for the duration of symptoms, time of occurrence, level of intensity, and types of symptoms. For some women, the pain and bleeding lasted longer than expected (several weeks or even months), and they were looking for community advice. MToP efficacy was a fundamental question during and after the MToP process. Users were wondering how to be sure that the procedure worked, and they expressed fear of failure. They also enquired about embryo expulsion: the appearance, at what moment it was supposed to be expelled, and how to know whether or not it had occurred. They also expressed concerns about surgical management of incomplete abortion. Postabortion sexual life was also of cardinal importance. Future fertility was also a concern: users were wondering when they could get pregnant again and when their menstrual periods would return to normal.

The need for psychological support was quite common after medical abortion. During the procedure, users reported feelings of loneliness. After the procedure, some users mentioned difficulties in overcoming the event, sometimes for a long time.

Overall, 5.4% of the 553 encountered difficulties (n=30) highlighted a lack of health care support during and after the procedure. Users reported that they were looking for health care providers who did not judge them and who could provide them with an environment of mutual trust.

## Discussion

### Principal Findings

The results of this exploratory study found that infodemiology could help to collect French women’s feedback about their MToP experiences. The results showed that women used social media to share their experiences, offer and find support, and provide and receive information regarding medical abortion. The extent of the need for information during and after the MToP procedure suggested that there is still room for improvement.

In the context of shared decision-making in medicine, where both the patient and physician contribute to the process and agree on treatment decisions, the relationship between women and health professionals is crucial and needs to be built up, including beyond the MToP act. To extend this connection, health care professionals can already rely on tools such as leaflets, institutional websites, or mobile apps (eg, chatbots) to answer additional questions from women. This ensures high-quality, standardized, and reachable information. In this study, the need for information did not necessarily mean a lack of information. The discovery of an unintended pregnancy, the idea of terminating it, the fear of stigmatization, and facing medical terms for an unprecedented situation can generate anxiety and ultimately difficulties for the woman in integrating the information provided by the health care professional. Independent of the counseling method, unbiased nondirective information should remain accessible (19% of posts sought advice) [27].

“Pain and bleeding” and “psychological experience” were also among the main topics. The psychological experience linked to the procedure was evoked at all stages of the process, from the pregnancy discovery to the MToP follow-up, showing that some women became apprehensive about this experience. Pain and anxiety were tightly associated; pain (physical and psychological) was mentioned in more than 1 out of 4 posts. This confirmed the previous evidence that some women needed timely counseling and education through this experience. Studies emphasize that listening to and accompanying women is essential [28]. The possibility of verbalizing physical pain could allow women to better bear the pain [29]. To satisfy the need for psychological support, the integration of the contact information of volunteer psychologists in the directories of health care professionals involved in the abortion process could facilitate access to psychological follow-up for women who wish to do so, instead of having to navigating the experience alone through the testimonies of online community members. This nonpharmacological individualized anxiety management could advantageously complete a pharmacological pain relief strategy.

Other encountered difficulties were reported (Figure 2). Fear of failure and its fallouts were mainly mentioned during and after abortion. Once the decision was made, there was an apparent need for reassurance about the success of their action. A timely counsel and education through a health care professional (eg, via telemedicine) or a community could meet this need. In the absence of a patient organization, the online community can offer adequate support. Indeed, pressure from the entourage and loneliness were mentioned in a small percentage of the posts. Abortion is not a neutral topic, and it can be either strongly encouraged or discouraged by the environment. This underlines the importance of meeting the woman alone to ensure the freedom of her choice [28]. When there is a language barrier, it is important to be able to call upon a professional interpreter [28]. In France, the law of March 20, 2017, protects women against disruption of access to abortion as a medical act and misinformation on the abortion procedure, particularly on the internet and social media [30]. Despite this law, no proceedings have led to a conviction so far. The persistent stigmatization of women who have recourse to abortion and the fear of the possible consequences of public exposure to a private and intimate situation may explain this, especially since misinformation is difficult to assess clearly and the law is still often misunderstood [31]. Nevertheless, a study conducted in 2019 in the planning center of a French hospital center (108 women) showed that 36% of women made their decision alone and 68% of women made their decision without difficulty (decision-making was assessed using the Decisional Conflict Scale) [32].

Concerning the psychological effects of MToP, the messages reporting regrets (including in the long term) underlined the importance of providing a caring listening ear (without bias or judgment) to women’s requests, and the importance of being able to offer women, when they feel the need, the possibility of psychological support.

### Study Strengths

The present results were obtained using data from social media. The use of social media to collect information has several advantages.

First, with, 2.3 billion users voluntarily sharing their data, experience, and outcomes, social media represent the new El Dorado to gather patient feedback [9,18]. Furthermore, the broad variety of social media and the long-term storage of public posts offer access to a large-scale data set allowing focus on specific topics, time periods, and locations. Indeed, the reactivity of social media facilitates carrying out analyses at a given moment and then over time. As such, these data make it possible to quickly measure the impact and acceptance of the implementation of a new health care procedure.

Second, the analysis of social media posts makes an important contribution by generating patient-centered perspectives from an underutilized data source. Our goal was to identify the direct experiences of MToP. Anonymity likely allowed women to express themselves without fear of recognition or judgment in this context. This alternative to in-hospital interviews helps to circumvent any form of white-coat bias [8,9].

Moreover, obtaining data from social media is facilitated by the low acquisition cost. This makes infodemiology an affordable methodology complementing standard clinical methods (ie, clinical studies or surveys), as it enables accessing a large data set while avoiding some of the intrinsic biases of standard methods.

Third, the median age of social media users reported in the study was consistent with the age for abortion in France [2].

Finally, the combined methodology of quantitative analysis and qualitative examination enabled robust characterization of topics, as previously described in peer-reviewed papers. This proven study type helps to give a voice to women experiencing MToP with limited background noise on this topic.

### Study Limitations

First, our study is subject to the inherent limitations of all infodemiology studies. One of these limitations results from the fact that, despite an abundant amount of data available, worldwide regulation prevented us from extracting posts from private forums/groups or those that are exchanged directly between users. Moreover, not all social media users are active. van Mierlo et al [33] estimated that approximately 90% of social media users are observers and do not actively participate in content creation; only 9% contribute sparingly and 1% create most of the content.

Another limitation is the variability of the level of contribution according to age (young people express themselves more than other age groups), gender (women express themselves more than men), country, socioprofessional class, and other factors [9,34]. In our study, as the data collected via social media were not representative of the population, there is a limitation in generalizing the findings to the whole French population of women who have experienced MToP [35].

Since the data were issued from the internet, our study could also present recall bias. Social media users tend to more frequently verbalize negative rather than positive experiences (ie, recall bias). This could lead to an overrepresentation of negative observations related to MToP in our study. It should also be noted that data published on social media could be deleted or modified, limiting the reproducibility of the results. The quality of the data collected was very heterogeneous and varied among social media users. Verifying the accuracy of published data is challenging due to the anonymity offered by social media. Content bots or users pretending to be others could have created some of the analyzed content.

Moreover, our analysis was based on the spontaneous testimonies of social media users on a single topic of interest and according to their feelings (subjectivity). The media or influential people could direct the discussions and encourage a peak of comments at a given moment (eg, the change of legislation around abortion in the United States).

Finally, due to variations among clinical practices and cultural differences, the conclusions of our study are not reproducible in different countries and regions.

### Conclusion

This exploratory study showed the added value of infodemiology. Applied to medical abortion, the results indicate that French women who underwent an MToP used social media to document their experiences, offer and find support, and provide and receive information regarding the procedure. This suggests that there is still room for improvement during and after the process, particularly in providing women with the opportunity to be properly informed, be listened to, and express themselves.

## Appendix

Multimedia Appendix 1 Keywords for data extraction.

Multimedia Appendix 2 Data extraction filters.

## Abbreviations

BTM: biterm topic model
MToP: medical termination of pregnancy
TF-IDF: term-frequency inverse document frequency
XGBoost: extreme gradient boosting

## Data Availability

According to French law, the data sets can only be shared through controlled access. Please contact Kap Code for any questions.
