# Supplementary figures and images for: Experiences of Women With Medical Abortion Care Reflected in Social Media (VEILLE Study): Noninterventional Retrospective Exploratory Infodemiology Study
Source: JMIR Infodemiology. 2024 May 2;4:e49335. doi: 10.2196/49335 (PMC11099808; doi:10.2196/49335)

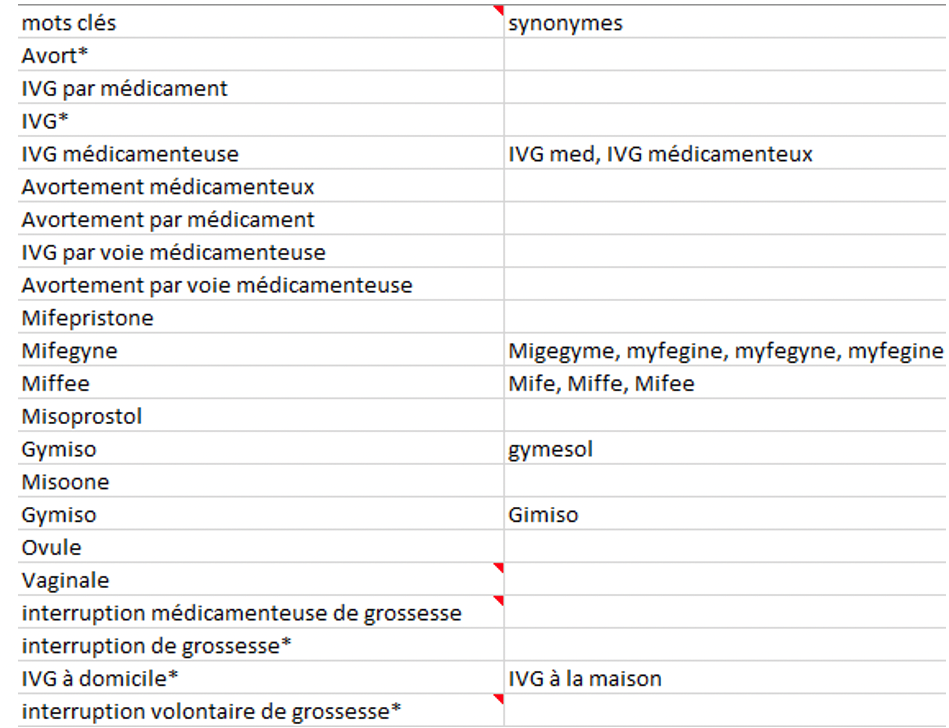

Supplement: Multimedia Appendix 1 [file infodemiology_v4i1e49335_app1.png]

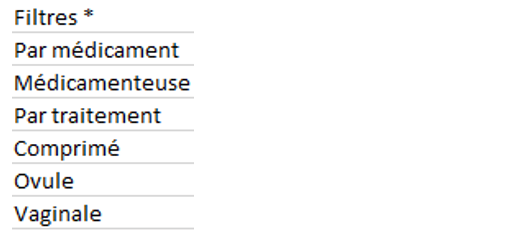

Supplement: Multimedia Appendix 2 [file infodemiology_v4i1e49335_app2.png]
